# Modeling the impact of the difference in cross-protection data between a human papillomavirus (HPV)-16/18 AS04-adjuvanted vaccine and a human papillomavirus (HPV)-6/11/16/18 vaccine in Canada

**DOI:** 10.1186/1471-2458-12-872

**Published:** 2012-10-13

**Authors:** Michele Kohli, Donna Lawrence, Jennifer Haig, Andrea Anonychuk, Nadia Demarteau

**Affiliations:** 1 OptumInsight, Health Economics and Outcomes Research, 5500 North Service Rd, Burlington, ON, L7L 6W6, Canada; 2 GlaxoSmithKline Biologicals, Global Vaccines Development, Avenue Fleming, 20, 1300, Wavre, Belgium

**Keywords:** Human papillomavirus, Cervical cancer, Vaccine, Cost-effectiveness, Markov model, Canada

## Abstract

**Background:**

In Canada, two vaccines that have demonstrated high efficacy against infection with human papillomavirus (HPV) types −16 and −18 are available. The HPV-6/11/16/18 vaccine provides protection against genital warts (GW) while the HPV-16/18 vaccine may provide better protection against other oncogenic HPV types. In this analysis, the estimated clinical and economic benefit of each of these vaccines was compared in the Canadian setting.

**Methods:**

A Markov model of the natural history of HPV infection among women, cervical cancer (CC) and GW was used to estimate the impact of vaccinating a cohort of 100,000 12-year-old females on lifetime outcomes and healthcare system costs (no indirect benefit in males included). A budget impact model was used to estimate the impact of each vaccine by province.

**Results:**

In the base case, vaccination with the HPV-16/18 vaccine was predicted to prevent 48 additional CC cases, and 16 additional CC deaths, while vaccination with the HPV-6/11/16/18 vaccine was predicted to prevent 6,933 additional GW cases. Vaccination with the HPV-16/18 vaccine was estimated to save 1 additional discounted quality adjusted life year (QALY) at an overall lower lifetime cost to the healthcare system compared to the HPV-6/11/16/18 vaccine (assuming vaccine price parity). In sensitivity analyses, the HPV-6/11/16/18 vaccine was associated with greater QALYs saved when the cross-protection efficacy of the HPV-16/18 vaccine was reduced, or the burden of GW due to HPV-6/11 was increased. In most scenarios with price parity, the lifetime healthcare cost of the strategy with the HPV-16/18 vaccine was predicted to be lower than the HPV-6/11/16/18 vaccine. In the probabilistic sensitivity analyses, the HPV-16/18 vaccine provided more QALY benefit than the HPV-6/11/16/18 vaccine in 49.2% of scenarios, with lower relative lifetime costs in 83.5% of scenarios.

**Conclusions:**

Overall, the predicted lifetime healthcare costs and QALYs saved by implementing each of the vaccines are similar. Vaccination with the HPV-16/18 vaccine is expected to be associated with reduced CC disease morbidity and mortality compared to vaccination with the HPV-6/11/16/18 vaccine. Differences in these outcomes depend on the extent of cervical disease prevented by cross-protection and the burden of GW caused by HPV-6/11.

## Background

Fifteen of the approximately 40 human papillomavirus (HPV) genotypes that infect the human anogenital area are known to be oncogenic [1]. While most HPV infections are self-limiting and resolve within 24 months, persistent HPV infection with an oncogenic type is the predominant cause of cervical cancer [2], the third most common cancer in women worldwide [3]. HPV types −16 and −18 account for approximately 70% of cervical cancer cases [4], while other oncogenic HPV types, including HPV-31, -33, -35, -39, -45, -51, -52, -56, -58, -59, -66 and −68, are involved in the remainder[5]. Of note, HPV-31 and −45 account for an additional 10% of cervical cancers worldwide [6] while HPV-16, -18 and −45 account for over 90% of adenocarcinomas [7]. Low-risk HPV genotypes, such as HPV-6 and −11, do not cause cancer but can lead to genital warts and low-grade pre-cancerous lesions [8].

In Canada, secondary prevention of cervical cancer through opportunistic and organized screening has reduced the burden of disease relative to other countries [9]. However, despite screening, approximately 1,300 Canadian women are still diagnosed with cervical cancer and 370 die from the disease each year [10]. Vaccination to prevent HPV infection is regarded as an effective strategy for controlling HPV-related diseases. In 2007, the Canadian Immunization Committee (CIC) recommended that “school-based HPV vaccination of one female cohort be implemented in all Canadian provinces and territories” [1]. The CIC set goals to reduce cervical intraepithelial neoplasia (CIN) 2/3, cervical cancer incidence, and cervical cancer related deaths by 60% over the next 20 to 35 years and to increase vaccine coverage up to 90% within five years of introduction of the program. All provinces had introduced school-based immunization programs by the 2008/09 school year targeting one age group, ranging from grades 4 to 8 (Table 1). Five provinces also implemented temporary catch-up vaccination programs in older age groups (Table 1).

**Table 1:** Overview of provincial vaccination program policies and target population inputs for the budget impact analysis

**Province**	Publicly funded HPV vaccination females*	**Start of vaccination program†**	**Assumed target age groups modelled for 2011/12 school year**	**Coverage**	**Size of target population**	**Number of females Vaccinated/Year**
British Columbia	Grade 6	2008-09	11 years	66% [12]^a^	24 027	15 858
	2008 – 2010 Only: Grade 9 Catch-up program [11] 2+1 Dosing strategy used					
Alberta	Grade 5	2008-09	10; 14; 15; 16 years	50%^a^	10 years: 19 489	10 years: 9 745
	Sept 2009 to June 2012 Only: Age 14–16 years Catch-up program [13]					
					14 years: 21 967	14 years: 10 984
					15 years: 22 563‡	15 years: 5 790
					16 years: 22 969 ‡	16 years: 3 060
Saskatchewan	Grade 6 or beyond with a date of birth no earlier than January 1, 1996. [14]	2008-09	11 years	85%^a^	5 652	4 760
Manitoba	Grade 6 [15]	2008-09	11 years	55%^a^	6 504	3 577
Ontario	Grade 8 [16]	2007-08	13 years	53% [17]^a^	74 048	39 246
Quebec	Grade 4 (2-dose in Grade 4 with booster in Grade 9) Grade 9 catch-up [18]	2008-09	9 years	76% [19]	9 years: 40 099	9 years: 30 476
			14 years		14 years: 46 756	14 years: 35 534
Newfoundland and Labrador	Grade 6[20] 2008–10 Only: Grade 9 catch-up cohort	2007-08	11 years	83%^a^	2 710	2 249
New Brunswick	Grade 7,	2008-09	12 years	85%^a^	4 080	3 468
	2008–09 Only: Grade 8 catch-up [21]		13 years (2008 Only)			
Nova Scotia	Grade 7 [22]	2007-08	12 years	80% [17]^a^	5 192	4 154
Prince Edward Island	Grade 6 [23]	2007-08	11 years	80% [17]^a^	723	578

* Women in the catch-up program are assumed to receive the same number of doses as those in the primary target population. Unless stated that a 2+1 dosing schedule is recommended, the provinces have adopted the recommended 3-dose schedule for the vaccines.

† The catch-up programs may have been initiated after the primary vaccination program began or implemented in selected school years only. For this analysis, only catch-up programs in the 2011/12 school year were modelled.

‡ In order to determine the number of women eligible for vaccination, the budget impact model calculates the number of women vaccinated since the implementation of the vaccination program in each province. Although there are expected to be 22 563 15-year olds and 22 969 16-year olds in Alberta in 2011, 10 983 and 16 849 respectively are expected to be vaccinated prior to the 2011/12 school year. Therefore, only 11 580 15-year olds and 6 120 16-year olds are eligible for the catch-up program targeting these age groups.

^a^ Coverage estimated by local experts unless otherwise specified.

Two HPV vaccines are currently available in Canada: a HPV-6/11/16/18 vaccine (*Gardasil*®, developed by Merck) has been licensed since July 2006, and a HPV-16/18 AS04-adjuvanted vaccine (*Cervarix*^®^, manufactured by GlaxoSmithKline), was approved in February 2010. Both vaccines provide excellent protection against HPV-16 and −18 and their related cervical outcomes. The HPV-6/11/16/18 vaccine is a quadrivalent vaccine that also provides protection against non-oncogenic HPV types −6 and −11 which can cause genital warts and CIN1. The HPV-16/18 AS04-adjuvanted vaccine has a proprietary AS04 adjuvant system which has been shown to enhance humoral and B cell responses compared to the same antigens adjuvanted with aluminium [24].

Cross-protection is the ability to prevent infection with oncogenic HPV types not contained in the vaccine. In clinical trials, the HPV-16/18 AS04-adjuvanted vaccine provided 100% (96.1% CI, 82.2 to 100) cross-protection against CIN2+ caused by HPV-31/45 and 68.4% (96.1% CI, 45.7 to 82.4) efficacy against CIN2+ caused by the 10 most common non-vaccine oncogenic HPV types (−31, -33, -35, -39, -45, -51, -52, -56, -58, -59) in an HPV-naïve population [25,26]. In a separate trial of HPV-naïve young women, the HPV-6/11/16/18 vaccine reduced CIN2/3/adenocarcinoma in situ (AIS) associated with HPV-31/45 by 58.7% (95% CI, 14.1 to 81.5) and CIN2/3/AIS associated with the same 10 HPV types by 32.5% (95% CI, 6.0 to 51.9) [27]. The majority of non-16/18 cervical cancers and CIN2/3 precancerous lesions, plus a proportion of CIN1 cases, are caused by these non-vaccine oncogenic HPV types. Therefore, greater cross-protection should translate into additional cases of CIN1, CIN2/3 and cervical cancer prevented. A dose of the HPV-6/11/16/18 vaccine is recommended to be administered at months 0, 2 and 6 [28], while a dose of the HPV-16/18 AS04-adjuvanted vaccine is recommended to be administered at 0, 1 and 6 months [29].

Economic modeling studies have consistently predicted that the adoption of HPV vaccination programs to prevent cervical cancer are economically attractive. In Canada, published analyses have found that HPV vaccination plus screening for cervical cancer is cost-effective compared to use of a screening program alone, with incremental cost-effectiveness ratios (ICERs) varying from approximately $18,000 – $32,000 per quality adjusted life year (QALY) gained in the base case analyses [30-33]. Only one of these published cost-effectiveness studies assessed the additional benefit associated with cross-protection against non-vaccine oncogenic HPV types. Anonychuk et al. [30] modeled a cohort of 100,000 12-year-old females vaccinated with the HPV-16/18 AS04-adjuvanted vaccine over a lifetime, using an economic model which included cross-protection and herd immunity. Results showed additional reductions in cervical cancer cases and deaths associated with cross-protection, and hence, demonstrated more economically attractive cost-effectiveness ratios.

A few published economic analyses have directly compared the HPV-16/18 AS04-adjuvanted and HPV-6/11/16/18 vaccines resulting in discordant conclusions. Those comparisons that did not consider the additional benefit of cross-protection concluded that the HPV-6/11/16/18 vaccine provides better value for money than the HPV-16/18 AS04-adjuvanted vaccine due to the additional costs saved and QALY gained by preventing genital warts attributed to HPV-6 and −11 [33-37]. The comparisons that included cross-protection have demonstrated that it has an important incremental impact on cancer outcomes and cost-effectiveness [38,39]. Demarteau et al. [40] examined the hypothetical difference in cross-protection efficacy needed for a vaccine with a profile similar to the HPV-16/18 AS04-adjuvanted vaccine to be cost-effective when compared with a vaccine similar to the HPV-6/11/16/18 vaccine in the settings of France, Ireland and Italy. Their discounted results predicted that the HPV-16/18 AS04-adjuvanted vaccine would be more economically attractive than the HPV-6/11/16/18 vaccine when the former provided an additional 22% cross-protection efficacy against 10 HPV types. Jit et al. [41] examined the impact of both vaccines in the United Kingdom. Although they concluded that the HPV-16/18 AS04-adjuvanted vaccine prevented additional cervical cancer cases, the HPV-6/11/16/18 vaccine’s ability to prevent genital warts saved more QALYs and health care costs than additional cross-protection with the HPV-16/18 AS04-adjuvanted vaccine.

Calculation of the incremental costs and benefits of a new intervention relative to existing health care strategies allows determination of efficiency or value for money. Decision makers are also concerned about the intervention’s impact on their budget because a new technology may be efficient but not affordable. It is also important for decision makers to understand the absolute clinical impact of a new intervention within their population. Budget impact models are often constructed to help quantify the number of people eligible for a new intervention as well as the costs of that new intervention in a particular jurisdiction [42].

The first objective of this analysis was to estimate the relative clinical and economic benefit of the HPV-16/18 AS04-adjuvanted vaccine compared with the HPV-6/11/16/18 vaccine in the Canadian setting while considering cross-protection efficacy. The second objective was to look at the clinical and cost impact of implementing each vaccine within each of the 10 Canadian provinces.

## Methods

**Figure 1:**
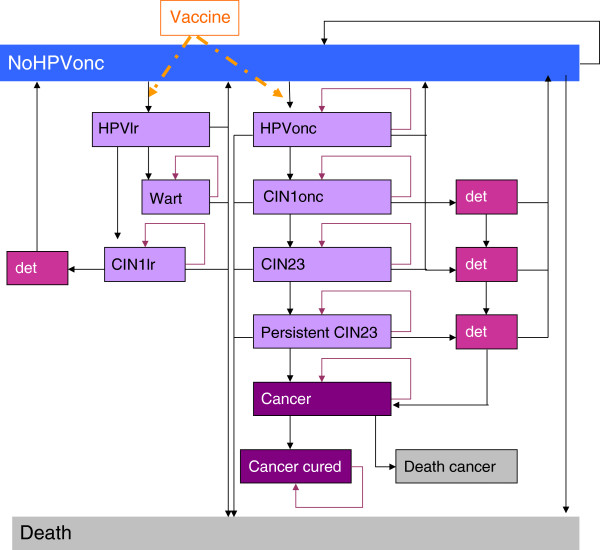
**Overview of the cohort model.** NoHPVonc: Women in this health state have no oncogenic HPV infection. HPVlr: Women in this health state have a low risk (non-oncogenic) HPV infection. HPVonc: Women in this health state have an oncogenic HPV infection. CIN1onc: Women in this health state have developed cervical intraepithelial neoplasia (CIN)1 due to an infection with an oncogenic HPV type. Wart: Women in this health state have genital warts. CIN1lr: Women in this health state have developed CIN1 due to an infection with a low risk HPV type. CIN23: Women in this health state have developed CIN2/3 due to an infection with an oncogenic HPV type. Det: represents women within each of the health states whose cervical disease is detected through screening.

This study used a previously published static Markov model that reproduced the natural history of oncogenic HPV with a one year cycle length [30,32]. Previous versions of this model simulated CIN1, CIN2/3, and cancer associated with oncogenic HPV types. This updated version includes non-oncogenic (low-risk) HPV infections, CIN1 disease due to non-oncogenic HPV infections, and genital warts (Figure 1) [40]. The model vaccine efficacy calculations, detailed by Debicki and colleagues [32], allow specification of the proportion of HPV types −16 or −18, non-vaccine oncogenic HPV types and HPV types −6 and −11 within all lesions, as well as vaccine efficacy by infection and lesion type.

**Figure 2:**
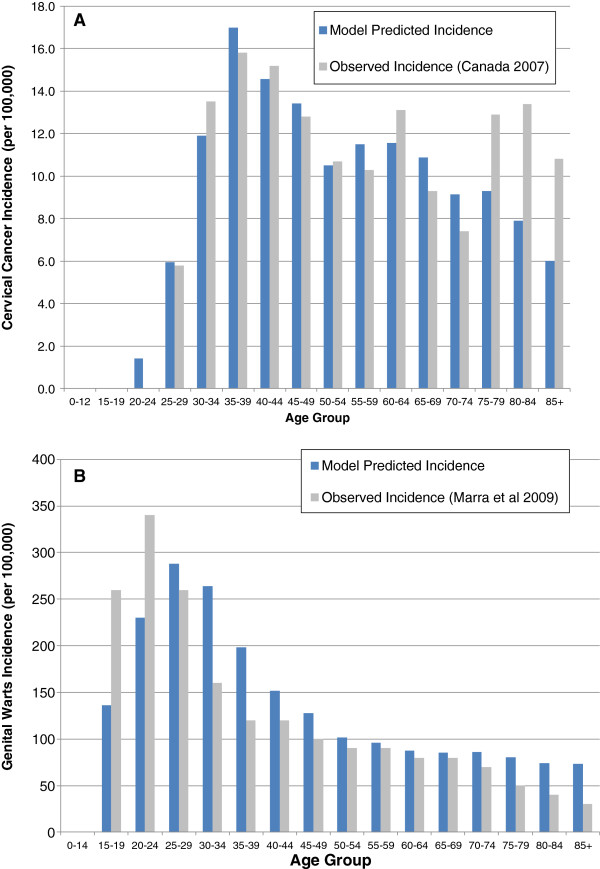
**Validation of the model.** Panel **A** shows the model predicted cervical cancer incidence per 100,000 women compared to reported data from Canada in 2007 [43]. Panel **B** shows the model predicted genital warts incidence compared to data reported by Marra et al. [44].

The model was developed in Microsoft^®^ Excel 2007 and simulates the effect of adding vaccination to the current screening program, where two cohorts of 100,000 12-year-old females were followed over a lifetime, one cohort vaccinated with the HPV-16/18 AS04-adjuvanted vaccine and the other with the HPV-6/11/16/18 vaccine. The model was previously calibrated to reproduce Canadian cervical cancer incidence and mortality, while keeping transition probabilities within pre-determined ranges [30,32]. The incidence of cervical cancer has been decreasing in older age groups, so the model calibration was updated as shown in Figure 2 to reflect the latest Canadian cervical cancer incidence data [43] and published genital warts incidence data [44].

Table 2 summarizes the base case model inputs. The model was parameterized using Canadian-specific screening, economic, and epidemiological data where available, as well as expert opinion. All events were costed from the perspective of the health care system in 2006 dollars. The incidence and cost per case of genital warts was obtained from analysis of an administrative databases in British Columbia, Canada [44]. The remaining data comes from the previously published Canadian analysis [30], with the exception of the distribution of HPV types within the health states displayed in Table 3. These distributions were updated to reflect a recent World Health Organization (WHO) review of Canadian data [45]. As vaccine coverage rates do not impact the cost-effectiveness ratios estimated with a static model, a coverage rate of 100% was assumed.

**Table 2:** Key base case cost-effectiveness model inputs

**Input parameters**	**Base case value**	**References**
**Vaccination**		
Duration of vaccine protection	Lifetime	Assumption
Age at Vaccination (years)	12	Assumption
Vaccine Coverage	100%	Assumption
Efficacy: HPV-16/18 (both vaccines)	98.0%	[26,46-50]
HPV-16/18 AS04-adjuvanted Vaccine		
Efficacy: Non-vaccine oncogenic HPV types		
CIN1	47.7% (95% CI: 28.9%, 61.9%)	[51]
CIN2+	68.4% (95% CI: 45.7%, 82.4%)	[25]
HPV-6/11/16/18 vaccine Efficacy: Non-vaccine oncogenic HPV types		[27]
CIN1	23.4% (95% CI: 7.8%, 36.4%)	
CIN2+	32.5% (95% CI: 6.0%, 51.9%)	
HPV-6/11/16/18 Vaccine Efficacy: HPV 6/11	98.0%	[50]
**Screening**		
Screening Coverage		[52]
Age Range	18 to 69 years	
Regular (once/ 3 years)	70%	
Irregular (ages 25, 40, 50)	18%	
Never	12%	
CIN1 detected (sensitivity)	42%	[44,52,53]
CIN2/3 detected (sensitivity)	55%	[44,52,53]
% Positive pap Smear	5%	[44,52,53]
**Cost (2006 CAD)**		
HPV-16/18 AS04-adjuvanted vaccine Cost (per dose)	$110.97	Assumption
HPV-6/11/16/18 vaccine Cost (per dose)	$110.97	Assumption
Genital Warts treatment (per episode)	$207.00	[44]
Cytology Test	$57.00	[30,33,54]
Colposcopy and biopsy	$150.00	[30,33,54]
CIN1 treatment and follow-up*	$843.00	[30,33,54]
CIN2/3 treatment and follow-up*	$1 414.00	[30,33,54]
Cervical cancer stage 1	$11 915.00	[30,33,54]
Cervical cancer stage 2	$18 851.00	[30,33,54]
Cervical cancer stage 3	$18 851.00	[30,33,54]
Cervical cancer stage 4	$25 759.00	[30,33,54]
**Utility**		
No HPV, HPV Infection	1, 1	
CIN1 detected	0.987	[55-58]
CIN2/3 detected	0.991	[55-58]
Cancer treated	0.727	[55-58]
Cancer cured	0.938	[55-58]
Genital Warts	0.980^†^	[59]

* Cost calculated assuming that 50% of CIN1 and 100% of CIN2/3 are treated. Follow-up after CIN1/2/3 assumed to include 2 additional cytology tests and colposcopies for all patients.

† Genital warts assumed to cause a decrement of 0.8 in utility across 3 months.

**Table 3:** Assumed HPV distributions for each model health state for the base case and sensitivity analyses

**Health state**	**HPV genotype**	**Canadian data**	**References**	**Base case estimates †**	**North America data**	**Sensitivity analysis estimates‡**	**References**
	16/18	24.9%	[45]	24.9%	25.6%	22.0%	[60]
CIN1	CP*	33.6%		33.6%	67.5%	57.0%	
	6/11	4.8%		4.8%	7.6%	6.0%	
	Other	6.9%		36.7%	17.8%	15.0%	
	16/18	56.2%	[45]	56.2%	55.2%	50.0%	[60]
CIN2/3	CP*	32.9%		32.9%	49.6%	45.0%	
	Other	2.5%		10.9%	5.6%	5.0%	
	16/18	74.3%	[45]	74.3%	76.5%	85.0%	[60]
Cancer	CP*	18.3%		18.3%	13.6%	15.0%	
	Other	2.2%		7.4%	1.0%	0.0%	
Genital Warts	6/11		[61]	76.2%		76.2%	[61]

* CP: Cross-protection – oncogenic HPV types affected by the vaccine: HPV-31,-33,-35,-39,-45,-51,-52,-56,-58,-59.

† The proportion in the other HPV category was increased as required so that all cervical outcomes were associated with an HPV infection.

‡ The sum of proportions of all HPV infections were equal to more than 100% for CIN1 and CIN2/3 lesions due to multiple HPV infections. For modelling purposes, the values in each HPV category were proportionally reduced so that sum of all HPV types equals 100% .

CC – Cervical cancer.

CIN – Cervical Intraepithelial Neoplasia.

HPV – Human papillomavirus.

A 98% vaccine efficacy against HPV types 16 and 18 was used based on the latest results from each vaccine’s clinical trials [25,27,47,49,50,62-66]. For the HPV-16/18 AS04-adjuvanted vaccine, 47.7% (96.1% CI: 28.9- 61.9) and 68.4% (96.1% CI: 45.7-82.4) cross-protective efficacy against CIN1+ and CIN2+, respectively, was demonstrated for the 10 most frequent non-vaccine oncogenic HPV types −31, -33, -35, -39, -45, -51, -52, -56, -58, and −59 in clinical trials [25]. For the HPV-6/11/16/18 vaccine, 23.4% (95% CI: 7.8 to 36.4) and 32.5% (95% CI: 6.0-51.9) cross-protective efficacy against CIN1+ and CIN2+ respectively for the same 10 non-vaccine oncogenic HPV types [27] was demonstrated in clinical trials. These efficacy values come from two independent studies that report efficacy values for an HPV-naïve population in a similar manner for the same HPV types. In this analysis, the observed reduction in CIN1+ was input as efficacy against CIN1, while the observed reduction in CIN2+ was input as efficacy against CIN2/3 and cervical cancer outcomes. A 98% vaccine efficacy against HPV types −6 and −11 was assumed for the HPV-6/11/16/18 vaccine based on clinical trial data [49,50]. This analysis assumed life-long protection against all HPV types, including cross-protection against non-vaccine types. Both of the vaccines were assumed to cost $100 per dose plus an administration fee of $10.97 [67], and it was assumed that 3 doses were given for both [28,29]. For the cost-effectiveness analysis, it was assumed that the entire cohort received all doses (100% coverage). Since this model is a static cohort model, it does not estimate the impact of reduced transmission of virus from women to men and any accompanying indirect benefit to men.

The lifetime number of CIN lesions, cervical cancer cases, cervical cancer related deaths, genital warts cases, QALYs, and costs were determined for each cohort. An ICER (cost per QALY gained) was calculated to compare the costs and outcomes of the two vaccines. A discount rate of 3% was applied to both costs and outcomes.

A number of sensitivity analyses were conducted by varying inputs assumed to impact the relative value of the two vaccines. The HPV-6/11/16/18 vaccine’s price per vaccine dose was varied to determine the point at which both vaccines would be predicted to have equivalent lifetime costs to the health care system. In a series of two-way sensitivity analyses, the efficacy of each vaccine against CIN2+ outcomes associated with non-vaccine oncogenic types was varied using the confidence intervals from the clinical trials. In addition, alternative WHO data for the continent of North America was used to populate the distribution of HPV types within cervical health states [68,69]. The overall impact of genital warts was tested with a series of one way sensitivity analyses varying the costs of genital warts (±25%), the quality of life impact (measured as a utility decrement) of genital warts (±25%; QALY decrement increased from 0.02 in base case to 0.041 [Maximum decrement observed in recent publications] [70,71]), and the incidence of genital warts (±10%; ±25%). The proportion of genital warts attributed to HPV types - 6 and - 11 was increased to 90% from the base case of 76%. Finally, simulation studies with transmission models have estimated that protecting females from HPV-6 / -11 may also reduce infection levels in males by as much as 90% over the next 70 years due to herd immunity [72]. Consistent with those predictions, Donovan et al. report an observed decrease in genital warts of 39% (95% CI 33–46; p trend <0.0001) in males aged 12 – 26 years in Australia since implementation of the quadrivalent vaccine [73]. As there has been no corresponding decrease in older males and very few males have received the vaccine, the authors attribute this decrease in young males to protection via herd immunity. In Sweden, however, Leval et al. reported decrease in genital warts amongst women but no decrease in men since implementation of an opportunistic program for females [74]. Although the model used for the current assessment includes females only, the impact of herd immunity on males was simulated by increasing overall genital warts incidence by multiplier of 2.0 or 2.2 and assuming quadrivalent vaccine efficacy to range from 20% to 90% of the cases that would normally be seen in men.

Multivariate probabilistic sensitivity analyses were conducted to explore the combined effect of parameter uncertainty using @Risk software (Palisade Corporation, Ithaca, New York, USA). Distributions were assigned to transition probabilities, vaccine effectiveness, proportion of outcomes (genital warts, CIN and cancer) attributed to each HPV type, screening effectiveness, costs, utilities using normal distribution (limited from 0–1 for transition probabilities) when confidence intervals were available, otherwise, a uniform distribution was assigned ranging from 25% below and above base case value (Table 4). In total, 10,000 samples were generated from the assigned distribution.

**Table 4:** Distributions used for probabilistic sensitivity analyses

**Variable names**	**Distribution**	**Distribution parameters**
**Probability of transitioning between health states**		
HPVOnc to CIN1	Normal^*^	0.076 (S.D. 0.009) [75]
HPV low risk to CIN1	Normal^*^	0.036 (S.D.0.005) [76,77]
CIN1 low risk regression	Normal^*^	0.5 (S.D. 0.145) [77]
CIN1 Onc Cured	Normal^*^	0.5 (S.D. 0.145) [76-78]
CIN1 Onc to CIN2/3 progression	Normal^*^	0.13 (S.D. 0.021) [76,78]
CIN2/3 Cured	Normal^*^	0.5 (S.D.0.058) [76,77]
HPV Onc regression	Uniform^†^	0.375 - 0.625[79]
HPVOnc to CIN2/3 progression	Uniform^†^	0.008 - 0.013 (assumption)
HPV Low risk regression	Uniform^†^	0.218 - 0.363 (assumption)
Genital Wart resistent	Uniform^†^	0.188 - 0.313[80]
Proportion CIN1 Onc detected and treated	Uniform^†^	0.375-0.625[76]
CIN1 treatment success	Uniform^†^	0.95 -1[76]
CIN2/3 progress to cancer	Uniform^†^	0.045 - 0.075 (assumption)
Proportion CIN2/3 detected and treated	Uniform^†^	0.9 -1 (assumption)
CIN2/3 treatment success	Uniform^†^	0.9-1 (assumption)
Cervical cancer to death	Uniform^†^	0.056 - 0.094[81]
Cervical cancer to cured	Uniform^†^	0.184 - 0.307[81]
**Utility data**		
No HPV	Fixed (1)	1 [57,58]
HPV	Fixed (1)	1 [57,58]
Death	Fixed (0)	0
Genital Wart	Uniform^‡^	0.015 - 0.025 [58,82]
CIN1 detected	Uniform^‡^	0.010 - 0.016 [57,58]
CIN2/3 detected	Uniform^‡^	0.007 - 0.012 [57,58]
Cancer	Uniform^‡^	0.205 - 0.341 [57,58]
Cancer cured	Uniform^‡^	0.047 - 0.078 [57,58]
**Screening effectiveness**		
CIN1 detected	Normal^Â§^	0.422 (S.D. 0.045) [81]
CIN2/3 detected	Normal^Â§^	0.554 (S.D. 0.045) [81]
Percentage estimated positive Pap smear	Uniform^‡^	0.035 - 0.059 (expert opinion)
**Vaccine effectiveness**		
HPV-16/18 AS04-adjuvanted vaccine efficacy against HPV-16/18	Normal (Mean: 98%; SD: 0.022)	0.98 (S.D. 0.022) [26,48]
HPV-6/11/16/18 vaccine efficacy against HPV-16/18	Normal (Mean: 98%; SD: 0.022)	0.98 (S.D.0.022) [43,50,83]
HPV-16/18 AS04-adjuvanted vaccine efficacy against other HPV onc	Normal^||^	0.48 (S.D. 0.083) [25]
HPV-6/11/16/18 vaccine efficacy against other HPV onc	Normal^||^	0.23 (S.D. 0.072) [27]
HPV-16/18 AS04-adjuvanted vaccine efficacy against CIN1 and other	Normal^||^	0.48 (S.D. 0.083) [25]
HPV-6/11/16/18 vaccine efficacy against CIN1 and other	Normal^||^	0.23 (S.D. 0.0715) [27]
HPV-16/18 AS04-adjuvanted vaccine efficacy against CIN2+	Normal^||^	0.68 (S.D. 0.092) [25]
HPV-6/11/16/18 vaccine efficacy against CIN2+	Normal^||^	0.33 (S.D. 0.115) [27]
HPV-6/11/16/18 vaccine efficacy against HPV-6 and −11	Normal^||^	0.98 (S.D. 0.065) [49,50]
**HPV type distributions**		
Proportion of HPV Onc	Uniform^†^	0.585 - 0. 975 [84]
Proportion of HPV-6/11 among warts in Canada	Uniform^†^	0.572 - 0.953 [84]
Proportion of HPV-16 and −18 among CIN1 in Canada	Uniform^†^	0.188 - 0.312 [84]
Proportion of HPV-16 and −18 among CIN2/3	Uniform^†^	0.437 - 0.729 [84]
Proportion of HPV-6 and −11 among CIN1	Uniform^†^	0.06 - 0.10 [84]
Proportion of HPV 10 types among CIN1	Uniform^†^	0.252 - 0.42 [84]
Proportion of HPV 10 types among CIN2/3	Uniform^†^	0.256 - 0.426 [84]
Proportion of HPV 10 types among CC	Uniform^†^	0.144 - 0.240 [84]
Proportion CIN1onc among CIN1 (other being CIN1LR)	Uniform^†^	0.51 - 0.85 [84]
**Cost data**		
HPV-16/18 AS04-adjuvanted vaccine vaccine	Uniform^‡^	$ 281 - $469 (assumption)

CC – Cervical cancer.

CIN – Cervical Intraepithelial Neoplasia.

HPV – Human papillomavirus.

Onc - oncogenic.

SD – Standard deviation.

* Normal distribution between 0 and 1 using as mean, the observed mean, and as standard deviation, 25% of the difference between the minimum and maximum value reported in the literature.

† Multiplied by a uniform distribution from 0.75 to 1.25 (with a maximum of 100%).

‡ Multiplied by uniform distribution from 0.75 – 1.25.

Â§ Normal distribution between 0 and 1 using as the mean: the observed mean and as the standard deviation 25% of difference of the confidence interval.

|| Normal distribution with a mean and standard deviation reported in clinical trials.

In order to customize the model to each of the Canadian provinces, a budget impact module that calculated the size of the target population and the cost of vaccination was constructed. It was linked to the cost-effectiveness model in order to predict lifetime costs and outcomes. The target population was based on the number of females in the assumed target age group for each province (Table 1) in the 2011/12 school year based on age-specific population data from Statistics Canada [85]. Two provinces, Alberta and Quebec, had catch-up programs in the 2011/12 school year and these populations were also modelled. In contrast to the cost-effectiveness analysis, coverage was not expected to be 100%: the number of females vaccinated within each of these age groups was calculated using expected coverage rates (Table 1). It was assumed that provinces employed either a 3 dose or a 2 + 1 dosing strategy (Table 1) based on current policies. In a 3 dose strategy, the aim is to deliver three doses during the school year, but it was assumed that vaccinees received an average of 2.7 doses due to imperfect coverage. In a 2 +1 strategy, the aim is to deliver two doses during the school year but it was assumed that vaccinees received an average of 1.95 doses. All vaccinated females were assumed to receive a follow-up booster dose 5 years later. The efficacy of a 2 + 1 dosing strategy is not officially approved for either vaccine and efficacy data for this strategy is not yet available from clinical trials. It was therefore assumed that the clinical efficacy of the 2 + 1 strategy was the same as that obtained with the 3 dose strategy.

The total annual cost from the perspective of the budget holder (i.e. the Department of Public Health) was calculated using the cost of vaccine purchase and administration. The net impact was calculated by subtracting the cost of providing all vaccinated females with the HPV-6/11/16/18 vaccine from the cost of providing all with the HPV-16/18 AS04-adjuvanted vaccine. The lifetime health care system costs associated with the HPV-16/18 AS04-adjuvanted vaccine plus cervical cancer screening were compared to those associated with the HPV-6/11/16/18 vaccine plus cervical cancer screening. The total cost of each strategy included the health care costs incurred by those receiving vaccination plus cervical cancer screening as well as those receiving cervical cancer screening only. The lifetime number of cervical cancer events experienced in the cohort of the assumed target population, including both vaccinated and unvaccinated individuals, was also calculated for each vaccine.

## Results

### Base case analysis

The results of the base case cost-effectiveness analysis for a cohort of 100,000 Canadian females across a lifetime time horizon are summarized in Table 5. Overall, vaccination with the HPV-16/18 AS04-adjuvanted vaccine was predicted to prevent 803 additional cases of CIN1, 651 additional cases of CIN2/3, 48 additional cases of cervical cancer, and 16 additional cervical cancer deaths. The HPV-6/11/16/18 vaccine on the other hand was predicted to prevent 6,933 more cases of genital warts in females. Since males are not modelled in this static simulation, the base case assumes no indirect benefit of vaccination in men due to reduced transmission of the virus in the population; reduction in genital warts cases amongst males is assumed to be zero. The HPV-16/18 AS04-adjuvanted vaccine was therefore associated with more life years gained than the HPV-6/11/16/18 vaccine, but the difference was offset to some degree when the utility decrements of the disease states were taken into account. Indeed, the HPV-16/18 AS04-adjuvanted vaccine was associated with 1 additional discounted QALY gained compared with the HPV-6/11/16/18 vaccine. Overall, the lifetime cost associated with treatment of cervical disease and genital warts outcomes from a health care system perspective was estimated to be lower with the HPV-16/18 AS04-adjuvanted vaccine than with the HPV-6/11/16/18 vaccine. In the base case, since the HPV-16/18 AS04-adjuvanted vaccine was predicted to be the lower cost strategy and to be associated with marginally more QALYs gained, it dominated the HPV-6/11/16/18 vaccine from a cost-effectiveness standpoint.

**Table 5:** Base case cost-effectiveness results for 100,000 women over a lifetime time horizon with 100% coverage

**Outcome**	**HPV-16/18 AS04-adjuvanted vaccine plus screening (A)**	**HPV-6/11/16/18 vaccine plus screening (B)**	**Difference in outcomes (A – B)**
**Undiscounted**			
CIN1 cases	8 217	9 020	−803
CIN2/3 cases	1 294	1 945	−651
Genital warts cases	9 688	2 755	6 933
Cervical cancer cases	113	161	−48
Cervical cancer deaths	38	54	−16
Life Years	7 163 635	7 163 268	367
Quality adjusted life years (QALY)	7 163 094	7 162 817	276
Lifetime cost of strategy	$127 212 309	$129 344 736	-$2 132 427
Incremental cost per QALY gained			A Dominates B
**Discounted outcomes**			
Life Years	2 982 064	2 981 994	70
QALY	2 981 855	2 981 854	1
Lifetime cost of strategy	$75 010 163	$75 693 270	-$683 107
Incremental cost per QALY gained			A Dominates B

A – Vaccine A: HPV-16/18 AS04-adjuvanted vaccine.

B - Vaccine B: HPV-6/11/16/18 vaccine.

QALY – Quality Adjusted Life Year.

Discount Rate – 3%.

The estimated clinical and cost impact of vaccinating with the HPV-16/18 AS04-adjuvanted vaccine and the HPV-6/11/16/18 vaccine using base case assumptions for each province is shown in Table 6. The model predicted that the use of the HPV-16/18 AS04-adjuvanted vaccine would prevent 0 (Prince Edward Island) to 32 (Quebec) additional cases of cervical cancer compared with the HPV-6/11/16/18 vaccine across the lifetime of the females targeted for vaccination in one school year due to the higher cross-protective efficacy observed in clinical trials. These estimates of cases saved included cervical cancers in both females who receive vaccination and those who did not. The predicted short-term costs represent those incurred from the perspective of the budget holder, and there is no difference between the vaccines since they are assumed to be priced equivalently. As described above, prevention of additional cervical disease was predicted to save more health care costs than prevention of genital warts, and this was reflected in the projections of long-term budget impact in Table 6.

**Table 6:** Projected total and net clinical and cost outcomes for 10 provinces in Canada

	**Alberta**	**British Columbia**	**Manitoba**	**New Brunswick**	**Newfoundland**	**Nova Scotia**	**Ontario**	**PEI**	**Quebec**	**Saskatchewan**
**Undiscounted lifetime cervical cancer cases**										
Vaccine A	223	71	23	8	6	11	269	2	208	11
Vaccine B	237	78	25	10	7	13	288	2	240	13
Difference (A- B)	−14	−8	−2	−2	−1	−2	−19	0	−32	−2
**Undiscounted short-term budget impact (CAD)**										
Vaccine A	8 862 430	3 431 536	1 071 737	1 039 079	673 843	1 244 617	11 758 847	173 180	14 284 003	1 426 186
Vaccine B	8 862 430	3 431 536	1 071 737	1 039 079	673 843	1 244 617	11 758 847	173 180	14 284 003	1 426 186
Difference (A-B)	0	0	0	0	0	0	0	0	0	0
**Discounted long-term budget impact (CAD)**										
Vaccine A	71 372 551	15 984 442	4 131 005	2 865 649	1 858 981	3 600 539	48 811 638	491 961	58 240 107	3 861 885
Vaccine B	72 015 197	16 091 773	4 155 215	2 890 712	1 874 203	3 630 560	49 128 834	495 873	58 720 943	3 894 102
Difference (A-B)	−642 645	−107 331	−24 210	−25 063	−15 222	−30 021	−317 196	−3 912	−480 836	−32 217

Short-term budget impact: Considers 1 year of vaccine purchase and administration costs.

Long-term budget impact: Considers lifetime costs to the health care system for the cohort of women eligible for vaccination in the 2011–12 school year. This includes both vaccinated and unvaccinated women.

CAD - Canadian dollars.

Vaccine A: HPV-16/18 AS04-adjuvanted vaccine.

Vaccine B: HPV-6/11/16/18 vaccine.

PEI – Prince Edward Island.

### Deterministic sensitivity analysis

The deterministic sensitivity analyses conducted with the cost-effectiveness model illustrate the importance of various inputs to the predicted relative clinical and economic impact of the two vaccines. Table 7 shows the impact of varying the degree of cross-protection that each vaccine provides. When the difference in cross-protection efficacy was reduced from the base case, the predicted profile of the HPV-6/11/16/18 vaccine relative to the HPV-16/18 AS04-adjuvanted vaccine improved. When the difference in cross-protection efficacy between the HPV-16/18 AS04-adjuvanted vaccine and the HPV-6/11/16/18 vaccine was assumed to be less than 16.5%, then the estimated costs saved and QALYs gained by preventing genital warts with the HPV-6/11/16/18 vaccine more than offset the benefit of any additional cervical disease predicted to be prevented by the HPV-16/18 AS04-adjuvanted vaccine. The HPV-6/11/16/18 vaccine therefore dominated the HPV-16/18 AS04-adjuvanted vaccine in these scenarios. When the difference in cross-protection efficacy between the HPV-16/18 AS04-adjuvanted vaccine and the HPV-6/11/16/18 vaccine was assumed to be 16.5% or 30.5%, then the QALYs gained by preventing cervical disease were predicted to be more than offset by the HPV-6/11/16/18 vaccine’s advantage in preventing genital warts. However, the projected lifetime costs associated with the HPV-16/18 AS04-adjuvanted vaccine were still lower in these two scenarios. When the HPV-16/18 AS04-adjuvanted vaccine was assumed to have a 35.9% or greater cross-protection efficacy (as in the base case), then the projected lifetime costs were lower and the estimated QALYs saved were greater compared with the HPV-6/11/16/18 vaccine. In other words, the HPV-16/18 AS04-adjuvanted vaccine was predicted to be better value for money than the HPV-6/11/16/18 vaccine. In all sensitivity analyses, the vaccine with the highest cross-protection efficacy was predicted to prevent the most cases of cervical cancer.

**Table 7:** Overview of the impact of varying the efficacy against CIN2+ outcomes associated with non-vaccine types

**Cross protection efficacy (%)**	**Difference in cross-protection efficacy**	**Difference in undiscounted lifetime cases of cancer**	**Difference in discounted lifetime healthcare cost**	**Difference in discounted lifetime QALY**	**Discounted incremental cost per QALY Gained**
	**(A – B)**	**(A – B)**	**(A – B)**	**(A – B)**	
A: 45.7; B: 51.9	−6.2	8	$628 335	−115	B Dominates A
A: 45.7; B: 32.5	13.2	−18	$28 868	−62	B Dominates A
A: 68.4; B: 51.9	16.5	−22	-$83 640	−52	$1 611
A: 82.4; B: 51.9	30.5	−42	-$530 970	−12	$43 499
Base Case: A: 68.4; B: 32.5	35.9	−48	-$683 107	1	A Dominates B
A: 45.7; B: 6.0	39.7	−52	-$771 520	10	A Dominates B
A: 82.4; B: 32.5	49.9	−67	-$1 130 437	41	A Dominates B
A: 68.4; B: 6.0	62.4	−83	-$1 483 495	73	A Dominates B
A: 82.4; B: 6.0	76.4	−102	-$1 930 825	113	A Dominates B

Cross-protection efficacy: Efficacy against CIN2/3+ outcomes attributed to non-vaccine oncogenic types.

A - Vaccine A: HPV-16/18 AS04-adjuvanted vaccine.

B - Vaccine B: HPV-6/11/16/18 vaccine.

QALY – Quality Adjusted Life Year.

Additional deterministic sensitivity analyses show the impact of reducing the price per dose of the HPV-6/11/16/18 vaccine, changing the assumed HPV distribution or altering the burden associated in genital warts (Table 8). Reducing the price per dose of the HPV-6/11/16/18 vaccine reduced the estimated discounted lifetime cost to the health care system associated with use of that vaccine. When the HPV-16/18 AS04-adjuvanted vaccine was priced at $100 per dose and the HPV-6/11/16/18 vaccine was priced somewhere between $97 and $98 per dose, the discounted lifetime health care costs associated with each vaccine were predicted to be equivalent. Due to the minimal difference in QALYs between the vaccines, when the price per dose of the quadrivalent is reduced from $98 to $97, the bivalent changes from dominating the quadrivalent to being a relatively poor value for money at an incremental cost per QALY of $145,773. Changing the data source for the HPV distribution from Canadian to North American values actually decreased the proportion of cases of cervical cancer attributed to the HPV types impacted by cross-protection. In this scenario, the HPV-16/18 AS04-adjuvanted vaccine was therefore predicted to prevent 41 more cases of cervical cancer than the HPV-6/11/16/18 vaccine, compared to 48 more cases in the base case. This relative reduction in benefit meant that the estimated QALYs saved using the HPV-6/11/16/18 vaccine were greater than that of the HPV-16/18 AS04-adjuvanted vaccine, although the lifetime cost associated with the HPV-16/18 AS04-adjuvanted vaccine was still lower. The remaining sensitivity analyses changed the burden associated with genital warts. The HPV-6/11/16/18 vaccine became relatively more attractive when the proportion of warts attributed to HPV-6/11 or the incidence of genital warts was increased, when the quality of life impact (disutility) associated with genital warts was increased or when protection against HPV-6/11 genital warts in men due to herd immunity was simulated. Changing the cost of treating genital warts impacted the predicted relative lifetime costs associated with the two vaccines, but not enough to give the HPV-6/11/16/18 vaccine an advantage. In all of these analyses, the HPV-16/18 AS04-adjuvanted vaccine was estimated to have an overall lower lifetime cost than the HPV-6/11/16/18 vaccine, except when the protection against HPV-6/-11 associated genital warts in males due to herd immunity is expected to be high (90%).

**Table 8:** Impact of varying vaccine price, genital warts inputs and HPV type distributions

**Description of sensitivity analysis**	**Difference in lifetime cases of cancer**	**Difference in discounted lifetime healthcare cost**	**Difference in discounted lifetime QALY**	Incremental cost per QALY gained*
	**(A – B)**	**(A – B)**	**(A – B)**	
Base Case	−48	-$683 107	1	A Dominates B
**Vaccine price**				
HPV-6/11/16/18 vaccine price decreased to $98 per dose	−48	-$83 107	1	A Dominates B
HPV-6/11/16/18 vaccine price decreased to $97 per dose	−48	$216 893	1	$145 773 B lower lifetime cost; A saves more QALYs
**HPV distribution**				
Changed to North American data	−41	-$760 577	−13	$59 359 A lower lifetime cost; B saves more QALYs
Proportion of 6 / 11 in genital warts increased to 90%	−48	-$496 855	−19	$26 015 A lower lifetime cost; B saves more QALYs
**Impact of herd immunity on male genital wart lesions**				
Double incidence of GW, assume herd immunity impact of 20%	−48	-$564 421	−8	$68 100 A lower lifetime cost; B saves more QALYs
Double incidence of GW, assume herd immunity impact of 30%	−48	-$461 020	−18	$25 388 A lower lifetime cost; B saves more QALYs
Double incidence of GW, assume herd immunity impact of 40%	−48	-$355 588	−28	$12 629 A lower lifetime cost; B saves more QALYs
Double incidence of GW, assume herd immunity impact of 50%	−48	-$248 084	−38	$6 481 A lower lifetime cost; B saves more QALYs
Double incidence of GW, assume herd immunity impact of 90%	−48	$203 554	−80	B Dominates A
Incidence of GW*2.2, assume herd immunity impact of 20%	−48	-$538 277	−10	$52 037 A lower lifetime cost; B saves more QALYs
Incidence of GW*2.2, assume herd immunity impact of 30%	−48	-$414 878	−22	$18 739 A lower lifetime cost; B saves more QALYs
Incidence of GW*2.2, assume herd immunity impact of 40%	−48	-$288 911	−34	$8 473 A lower lifetime cost; B saves more QALYs
Incidence of GW*2.2, assume herd immunity impact of 50%	−48	-$160 318	−46	$3 468 A lower lifetime cost; B saves more QALYs
Incidence of GW*2.2, assume herd immunity impact of 90%	−48	$381 563	−96	B Dominates A
**Genital warts incidence**				
+25%	−48	−449 523	−20	$22 485 A lower lifetime cost; B saves more QALYs
+10%	−48	−589 284	−7	$82 535 A lower lifetime cost; B saves more QALYs
−10%	−48	−777 456	10	A Dominates B
−25%	−48	−919 979	23	A Dominates B
**Genital warts cost**				
Increased to 125% of base case	−48	-$444 754	1	A Dominates B
Decreased to 75% of base case	−48	-$924 966	1	A Dominates B
**Genital warts disutility**				
Increased to 125% of base case	−48	-$683 107	−21	$32 749 A lower lifetime costs; B saves more QALYs
Decreased to 75% of base case	−48	-$683 107	24	A Dominates B
0.041, Drolet et al.	−48	-$683 107	−95	$7 170 A lower lifetime costs; B saves more QALYs

A – Vaccine A - HPV-16/18 AS04-adjuvanted vaccine.

B - Vaccine B: HPV-6/11/16/18 vaccine.

QALYs – Quality adjusted life years.

* A Dominates B – HPV-16/18 AS04-adjuvanted vaccine associated with lower lifetime costs to the healthcare system than HPV-6/11/16/18 vaccine; HPV-16/18 AS04-adjuvanted vaccine also saves more QALYs than HPV-6/11/16/18 vaccine.

### Probabilistic sensitivity analysis

**Figure 3:**
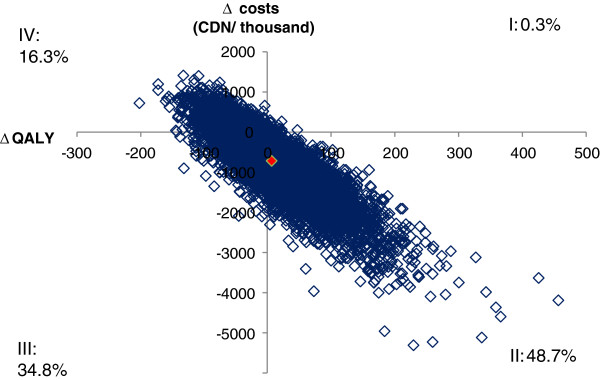
**Incremental cost effectiveness plane for vaccination of 100,000 12 year-olds with the HPV-16/18 AS04-adjuvanted vaccine compared with the HPV-6/11/16/18 vaccine across a lifetime time horizon (discounted).** Each dot on the graph represents the relative discounted costs (per 100,000 Canadian dollars (CDN)) and discounted quality adjusted life years (QALY) of one out of the 10,000 completed simulations. The difference in discounted costs and QALYs is calculated by subtracting the HPV-6/11/16/18 vaccine (Vaccine B) from the HPV-16/18 AS04-adjuvanted vaccine (Vaccine A) values (e.g. A – B). Quadrant I contains scenarios where the HPV-16/18 AS04-adjuvanted vaccine is associated with greater lifetime costs and more QALYs saved (0.3% of simulations). Quadrant II contains scenarios where the HPV-16/18 AS04-adjuvanted vaccine is associated with lower lifetime costs and more QALYs saved (C is dominant: 48.7% of simulations). Quadrant III contains scenarios where the HPV-6/11/16/18 vaccine is associated with greater lifetime costs and more QALYs saved (34.8% of simulations). Quadrant IV contains scenarios where the HPV-6/11/16/18 vaccine is associated with lower lifetime costs and more QALYs saved (G is dominant: 16.3% of simulations).

The results of the probabilistic sensitivity analysis are illustrated using the cost-effectiveness plane in Figure 3 where the result of each individual analysis is represented by a dot. In 0.3% of the replicates, the HPV-16/18 AS04-adjuvanted vaccine was predicted to have greater lifetime costs and greater QALY benefit than the HPV-6/11/16/18 vaccine (Quadrant I of figure). The HPV-16/18 AS04-adjuvanted vaccine was predicted to have lower lifetime costs and a greater lifetime benefit in 48.7% of scenarios (Quadrant II; the HPV-16/18 AS04-adjuvanted vaccine is dominant). The opposite was true in 16.3% of scenarios (Quadrant IV; the HPV-6/11/16/18 vaccine is dominant), while the HPV-6/11/16/18 vaccine was predicted to have greater lifetime costs and greater benefit in 34.8% of analyses. In other words, the QALY benefit was estimated to be greater for the HPV-16/18 AS04-adjuvanted vaccine in 48.9% of scenarios and for the HPV-6/11/16/18 vaccine in 51.1% of scenarios. There were more scenarios where the lifetime costs associated with the HPV-16/18 AS04-adjuvanted vaccine were estimated to be lower than that of the HPV-6/11/16/18 vaccine (83.5%).

## Discussion

This analysis presents a comparison of the relative clinical and economic benefit of the two HPV vaccines that are licensed for use in Canada using a static Markov model reproducing HPV disease in women. In the base case analysis, compared with the HPV-6/11/16/18 vaccine, the HPV-16/18 AS04-adjuvanted vaccine is associated with an overall greater reduction in cervical cancer and precancerous lesions, and a greater number of QALYs saved, for an overall lower lifetime cost to the health care system. Hence, under base case assumptions, the HPV-16/18 AS04-adjuvanted vaccine provides better value for money than the HPV-6/11/16/18 vaccine. Indeed, the difference in the discounted QALYs saved between the two vaccination strategies is small, and this difference is greatly impacted in the sensitivity analyses. The HPV-6/11/16/18 vaccine is associated with higher QALYs saved when the relative cross-protection benefit of the HPV-16/18 AS04-adjuvanted vaccine is reduced, the proportion of genital warts due to HPV-6/11 is increased, or the impact of genital warts on utility (the disutility) is increased. In all scenarios, except where the cross-protection benefit of the HPV-16/18 AS04-adjuvanted vaccine is assumed to be lower than the HPV-6/11/16/18 vaccine, vaccination with the HPV-16/18 AS04-adjuvanted vaccine is associated with an overall lower lifetime cost to the health care system when the vaccines are priced at parity. In the probabilistic sensitivity analyses, the HPV-16/18 AS04-adjuvanted vaccine provides more QALY benefit than the HPV-6/11/16/18 vaccine in 49.2% of scenarios, while it has lower relative lifetime costs in 83.5% of scenarios. The budget impact model analyses illustrate how the clinical and cost differences between the vaccines may manifest using estimated target populations in each of the Canadian provinces.

### Comparison to previously published analyses

Two other publications have compared the costs and benefits of the HPV-16/18 AS04-adjuvanted vaccine and the HPV-6/11/16/18 vaccine while considering the current cross-protection efficacy data from clinical trials. Capri and colleagues used a population model and estimated that the additional cross-protection benefit associated with the HPV-16/18 AS04-adjuvanted vaccine would prevent an additional 295 cases of cervical cancer in the Italian population compared with the HPV-6/11/16/18 vaccine [39]. They also concluded that the health care cost savings associated with preventing additional precancerous and cancerous lesions would offset the savings associated with preventing genital warts. Jit and colleagues [41] used a dynamic transmission model to estimate the impact of the vaccine in the United Kingdom looking at cervical cancer, genital warts and other HPV associated cancers. In their analysis, the costs and QALYs saved associated with preventing genital warts outweighed the costs and QALYs saved associated with preventing cervical cancer. The difference in the results between the British analysis described above and this Canadian analysis may be because the difference in cross-protection benefit was assumed to be smaller than in this analysis: Jit et al. [41] assumed 47.7% and 24.3% cross-protection efficacy for the HPV-16/18 AS04-adjuvanted vaccine and the HPV-6/11/16/18 vaccine respectively for all cervical outcomes. In addition, the incidence of genital warts was assumed to be more than double the incidence in our analysis. The United Kingdom study used to parameterize the model by Jit et al. [41] estimated annual genital warts incidence (new and recurrent cases) at 289/100,000 population [86], whereas the Canadian study used for this analysis estimated it at 126/100,000 [44]. Finally, Jit et al. used a higher discount rate (3.5% compared to 3.0% in our analysis) which will impact the costs accrued at older ages (e.g. cervical cancer treatment costs) more than those accrued at younger ages (e.g. genital warts treatment costs).

In a third publication, Demarteau and Standaert conducted analyses using the model in this publication adapted to France, Ireland, and Italy to determine the impact of cross-protection on the relative cost-effectiveness of a hypothetical bivalent and a hypothetical quadrivalent vaccine. The bivalent vaccine was assumed to prevent 95% of HPV types −16 and −18 infection while the quadrivalent prevent 95% of HPV type −16, -18, -6, and −11 infections. Both vaccines were assumed to provide lifetime protection against HPV-16, while efficacy against HPV-18 was assumed to wane after 10 years. With the quadrivalent vaccine, efficacy against HPV-6 and −11 was also assumed to wane after 10 years. The discounted incremental cost per QALY of the bivalent vaccine compared to the quadrivalent vaccine fell below a cost-effectiveness threshold of the gross domestic product (GDP) when the bivalent provided an additional 22% in cross-protection efficacy in France, 48% in Ireland, and 43% in Italy. The WHO suggests that interventions with an incremental cost per QALY below this threshold be considered as highly cost-effective [87].

### Limitations

This analysis is subject to limitations common to all decision analytic models in that it combines data from numerous sources, requires structural and data assumptions, and can be subject to certain biases. Given the uncertainty in epidemiological data, a number of simplifying assumptions were made. Co-infection with multiple oncogenic HPV types for example is not explicitly modeled. Patterns of HPV infection and cervical cancer screening practices were modeled based on average Canadian data and may therefore vary from actual practices in each of the provinces. The health economic model that generates estimates of lifetime costs and clinical impact in this analysis is a static Markov model reproducing disease burden in women, and as such does not account for the benefits associated with herd immunity of female vaccination on men. In terms of cervical cancer outcomes, the impact of vaccinating females may therefore be underestimated for both vaccines. We did not directly model outcomes in men who may benefit from a reduction in transmission of HPV-6/11 and an associated reduction in genital warts. We have attempted to simulate the impact of this in a number of sensitivity analyses, however, this indirect benefit can only be estimated with a dynamic model.

In this analysis, it was assumed that both vaccines provided lifetime protection, but the true duration of protection of the vaccines is not yet known. In a comparative clinical immunogenicity/safety study of the HPV-16/18 AS04-adjuvanted vaccine and the HPV-6/11/16/18 vaccine, the HPV-16/18 AS04-adjuvanted vaccine induced superior neutralizing antibody response compared to the HPV-6/11/16/18 vaccine for both HPV-16 and −18 in 18–45 year old women up to 24 months after first injection [88,89]. Although an immunological correlate of protection is not currently defined, differences in the magnitude of the immune responses between vaccines may represent determinants of duration of protection [88]. If one of the vaccines provided less than lifetime protection, the projected impact on lifetime cervical cancer cases would decrease while the lifetime cost associated with that vaccination strategy would increase. While we have attempted to identify two publications with comparable estimates of cross-protective efficacy for the 10 HPV types of interest, there is no single trial that directly compares the cross-protective efficacy of the bivalent and quadrivalent vaccine. Finally, cross-protection efficacy against the 10 HPV types is reported as a composite endpoint in clinical trials and is captured as such in our model. Type-specific cross-protection efficacy has not been reported for both vaccines; if efficacy varies by type then the overall cross-protection efficacy will depend on type-specific HPV prevalence which may vary by population. The combined data are the most similar across the two vaccines and also provide more robust estimates for the rarer outcomes such as CIN lesions.

HPV infection may cause cancers at other sites in women, including anal, vaginal, vulvar, and head and neck cancers, and these outcomes were not modeled in this analysis. Jit and colleagues [41] demonstrated in their United Kingdom analysis that accounting for the protection against the other HPV-related cancers does impact the relative value of the vaccines depending on assumptions about the efficacy of the vaccines in preventing HPV infections associated with these cancers. If both vaccines are assumed to have equivalent efficacy in preventing HPV infections leading to these outcomes, then their exclusion from the analysis does not impact the conclusion about the relative value of the HPV-16/18 AS04-adjuvanted vaccine and the HPV-6/11/16/18 vaccine. If one vaccine is assumed to provide superior protection against these other cancers, then the exclusion from the analysis will bias results.

## Conclusions

Based on our model, implementation of an HPV-immunization program in Canada using the HPV-16/18 AS04-adjuvanted vaccine is expected to be associated with a lower lifetime cost and a similar number of QALYs saved compared to the HPV-6/11/16/18 vaccine under base case assumptions. Overall, the HPV-16/18 AS04-adjuvanted vaccine reduced additional cervical cancer disease morbidity and mortality compared with the HPV-6/11/16/18 vaccine but had no impact on the morbidity associated with genital warts. From an economic perspective, the lifetime relative costs and QALYs saved by implementing each of the vaccines will depend on the assumptions about the extent of cervical disease caused by HPV types prevented by cross-protection, and the burden of genital warts caused by HPV-6/11. The results of this analysis may therefore differ in countries with a different portion of cervical disease attributed to HPV types impacted by cross-protection or a higher genital warts burden.

## Endnotes

*Cervarix*^®^ is a registered trade mark of the GlaxoSmithKline group of companies. *Gardasil*^®^ is a registered trade mark of Merck and Co., Inc.

## Abbreviations

AIS: Adenocarcinoma in situ; CC: Cervical cancer; CAD: Canadian dollars; CIC: Canadian Immunization Committee; CI: Confidence interval; CIN: Cervical intraepithelial neoplasia; CP: Cross-protection; GDP: Gross domestic product; HPV: Human papillomavirus; ICER: Incremental cost-effectiveness ratio; Onc: Oncogenic; PEI: Prince Edward Island; QALY: Quality adjusted life year; SD: Standard deviation; WHO: World Health Organization.

## Competing interests

MK, DL, JH are employees of OptumInsight, and OptumInsight was contracted by GlaxoSmithKline to collaborate on the research project and manuscript production. ND is an employee of GlaxoSmithKline Biologicals. AA was an employee of GlaxoSmithKline and is now an employee of Abbott Laboratories.

## Authors’ contributions

MK led the design of the analyses, the design of the budget impact analysis and drafted the manuscript. DL participated in the design of the study, performed the analysis, and assisted in drafting the manuscript. JH helped design and build the budget impact model, performed all of these analyses. ND conceived of the study, and participated in its design and coordination. ND created the original cost-effectiveness original model and AA populated the model with Canadian inputs. ND oversaw the probabilistic sensitivity analyses. All authors reviewed and approved the final manuscript.

## Pre-publication history

The pre-publication history for this paper can be accessed here:

http://www.biomedcentral.com/1471-2458/12/872/prepub
